# Is sexual misconduct training sufficient in the UK's medical schools: Results of a cross-sectional survey and opportunities for improvement

**DOI:** 10.1177/20542704231198732

**Published:** 2023-09-13

**Authors:** Tia Dowling, Sarah Steele

**Affiliations:** 1 Population Health Sciences Partnership, 2152 University of Cambridge, Cambridge, UK; 2 St Edmund's College, Cambridge, UK; 3 ThinkLab, University of Cambridge, Cambridge, UK; 4 Intellectual Forum, Jesus College, Cambridge, UK; 5 Department of Social and Political Sciences, Bocconi University, Milan, Italy

**Keywords:** medical education, sexual misconduct, sexual harassment training, freedom of information act, United Kingdom

## Abstract

**Objective:**

Sexual misconduct adversely affects the mental and physical health of millions of people each year and has been declared a global pandemic. Incidence in both educational and clinical settings remains high. In the last 5 years, the NHS spent over £4 million settling sexual misconduct-related claims. Effective prevention requires training across all stages of career, beginning in clinical school. Here, we explore training in the UK's medical schools to identify provision and areas for improvement.

**Design:**

Freedom of Information Act 2000 requests for data on training delivery and curricula content.

**Setting:**

34 public UK universities offering medical education

**Participants:**

not applicable

**Main Outcome Measures:**

Provision and delivery of training, mode of delivery, theme, and continuation of training.

**Results:**

All 34 universities responded. Twenty-two identified offering training. Seventeen made it compulsory. A review of curricula identified, however, only 18 did more just than mentioned sexual misconduct. Nine offered training more than once. Twelve did not offer training, of which three identified no plans to offer such training in the future. The most common delivery modes for training were workshops and lectures. The training was most often within the sexual health curriculum, disconnecting it from professionalism.

**Conclusions:**

There is no standardisation of sexual harassment training across the UK's public medical schools. Many future doctors will not have received relevant education when they assume posts in the NHS. Considering the magnitude of this issue and its critical connection to professionalism and collegiality, universities and professional bodies urgently should address this deficiency.

## Introduction

In 2017, the #MeToo movement reinvigorated discussions around the world about sexual misconduct, including sexual harassment, assault and other unwanted or uninvited behaviours of a sexual nature that affront, embarrass, harm, demean or intimidate an individual or group.^1^ Recognising the many physical and mental health impacts of sexual misconduct, international organizations emphasised it is a form of gender-based violence, acknowledging that it is so widespread, and its impacts so significant, that it is an issue demanding global attention.^2–5^ An urgent priority, it demands commitments from across societies and workplace to change social norms, as well as requiring reviews of structures, processes, and policies so as to prevent harassment, while also robustly addressing misconduct when it happens.^2–4^

Within medicine, various reports have been initiated by governments and professional organizations directed to ascertain the extent of sexual misconduct within healthcare settings and amongst the profession, while also exploring the adequacy of existing responses.^6–8^ Commentary following these reports note that harassment remains pervasive, has significant impacts on retention of clinical staff, especially women, and continues to be poorly addressed.^9^ Beyond the impact on individuals, there are wider health systems impacts with, for example Freedom of Information requests identifying that between 2018–2022, the National Health Service (NHS) in the United Kingdom (UK) paid GBP£4,020,231 in damages for sexual misconduct.^10^ Preventing misconduct is therefore a pressing issue for public health and health systems, but still training in the NHS has recently been criticised for not being comprehensive and insufficiently skills-focused.^11^

Indeed, in the UK, various bodies including the Professional Standards Authority, British Medical Association (BMA), and the General Medical Council (GMC)—the body that registers and licences medical practitioners in the UK and is engaged in quality assurance for medical education in UK universities—recognise that such behaviour is prevalent and that such forms of misconduct impact not only patient safety, but also creates cultures of mistrust and uncertainty amongst colleagues.^6,7,12^ Consequently, the GMC, like many similar professional bodies around the world, offer guidance to practitioners, along with rules, and recommends education and training.^8,12^ Tackling sexual misconduct is, then, connected to professionalism; encouraging calling out within healthcare to ensure quality and safety; doctors use of social media; and delivering on equality, diversity, and inclusion commitments.^12^

Quite notably, all four of these areas are pertinent to medical education, and thereby we expect that training should form part of the core curricula in these domains, even if presented briefly. In one study with healthcare educationalist and healthcare experts, over 80 percent identified that the gender concepts of “sexism” and “sexual harassment” are *essential* for medical and nursing students to learn about and understand.^13^ Indeed, as the future of the medical profession, students in clinical schools are key targets for interventions to reshape social norms and create a culture in medicine that recognise sexual harassment as poor behaviour that is incompatible with good medical practice.

The objective of this study was, therefore, to analyse the delivery of such training in medical schools in the UK. Here, we ask: to what this training is being wound out across the medical curricula in the UK? In what year/s is it presented? Where in the curricula is it presented? Does its presentation align with the domains in the GMC guidance? Our results are presented in order to guide medical educators both in the UK and beyond on the opportunities to better prepare tomorrow's doctors to understand their obligations and feel capable to identify and report sexual misconduct when they see it.

## Methods

Requests for data were made to all accredited medical schools in the UK using the Freedom of Information Act 2000 and the Freedom of Information Act (Scotland) 2002 (hereafter, referred to collectively as FOIA). FOIA allow individuals and organizations to request and obtain information from government bodies and other public sector organizations, with a view to promote transparency, accountability, and public engagement. Schools were identified through the GMC's list published on 15 November 2021,^14^ and of these, 34 were public bodies covered by the FOIA laws.^15,16^ One private medical school was sent a request but was not required to respond by law.

The request asked, “whether medical students receive any training on sexual harassment and violence in the professional context of being a doctor as part of their studies”. Definitions of misconduct like harassment and abuse were defined and clarified when requested. The request was designed to exclude training that focused on the clinical treatment of victims of sexual harassment or abuse. Follow-up questions were asked based on the initial response of ‘yes’ or ‘no’, to reduce the time it takes for schools to respond to the request. For schools answering ‘yes’, the next questions were designed to determine: 1) whether the training is compulsory or optional; 2) to request any training content used; and 3) to ascertain the percentage of students that have attended training. For schools answering ‘no’, they are asked whether they are actively considering harassment training for their medical students.

Data were collated and recorded in Microsoft Excel. The data obtained from the email responses were used to perform descriptive statistics and qualitative analysis for the presence of training, mode of delivery, theme of training, and duration of training across the course. University responses were categorized as: 1) having sexual harassment training in the context of being a doctor; 2) having good sexual harassment training in the context of being a doctor, or 3) having no training. This categorization was achieved through qualitative analysis of responses and training material, with “good training” defined as training that explicitly explored sexual harassment—that is, mention sexual harassment in a clear and detailed manner, leaving no room for confusion or doubt.

Notably, studies using FOIA requests are exempt from institutional ethics review processes as they are requests for data that are public by nature and the responses from the universities are publicly available in accordance with the relevant legislation.^17^ However, data in this study were anonymized for publication to avoid individual criticism of a school.

## Results

Responses were received from all GMC-approved public medical universities (*N *= 34). The University of Buckingham, the only private medical university in the UK, denied the request to voluntarily provide the requested information, stating that they do not have a legal obligation to answer.

Of the 34 responses, 65 percent (*N *= 22) identified that they offer sexual harassment training in the context of being a doctor. Of these, 18 percent (*N *= 4) identified only discussing general harassment, without identifying sexual harassment explicitly, and thus were reclassified. Only 53 percent (*N *= 18) universities therefore had some or good sexual harassment training.

We classified a response as “no training” when students would have to extrapolate or infer the relevance to sexual harassment, or where it was not mentioned at all. Following this classification, 47 percent (*N *= 16) had no training or training that did not specifically address sexual harassment directly. As an example, one response identified that it details the GMC guidance and sets out how to “behave well”, stating:

Accordingly, there are sections on treating colleagues fairly and with respect and maintaining trust with patients through respecting their dignity and privacy. It is axiomatic that sexual harassment and/or violence is incompatible with fairness, respect, and dignity.

While admirable, it is notable that sexual harassment is not covered directly, and it is expected that students will make the link between this expectation of good behaviour being incompatible with poor behaviour like sexual harassment, applying this to not only their own practice, but further connecting this explicitly to their colleagues.

Notably, three universities identified that students could take generalised consent and anti-harassment training in the wider university context, often offered by student unions or as part of residential arrival programmes, but these made no references to being a doctor and were disconnected entirely from clinical education and healthcare contexts. One university, for example, stated:

… ALL first-year students in the University who are accommodated in our residences, receive training on consent and definitions of sexual violence and harassment in Intro Week.

Another university stated:

[A] general training course on consent is mandatory for all… students living in on-campus residences.

While such consent and harassment training is desirable for all students in the higher education context, it is notably not connected to the profession, comprising of generalised content around consent in sexual relationships that sits outside the clinical context, and that does not consider the unique issues around patient care, medical professionalism and ethics, and calling out poor or unethical behaviour in medicine, where patient safety is a concern.

**Figure 1.**
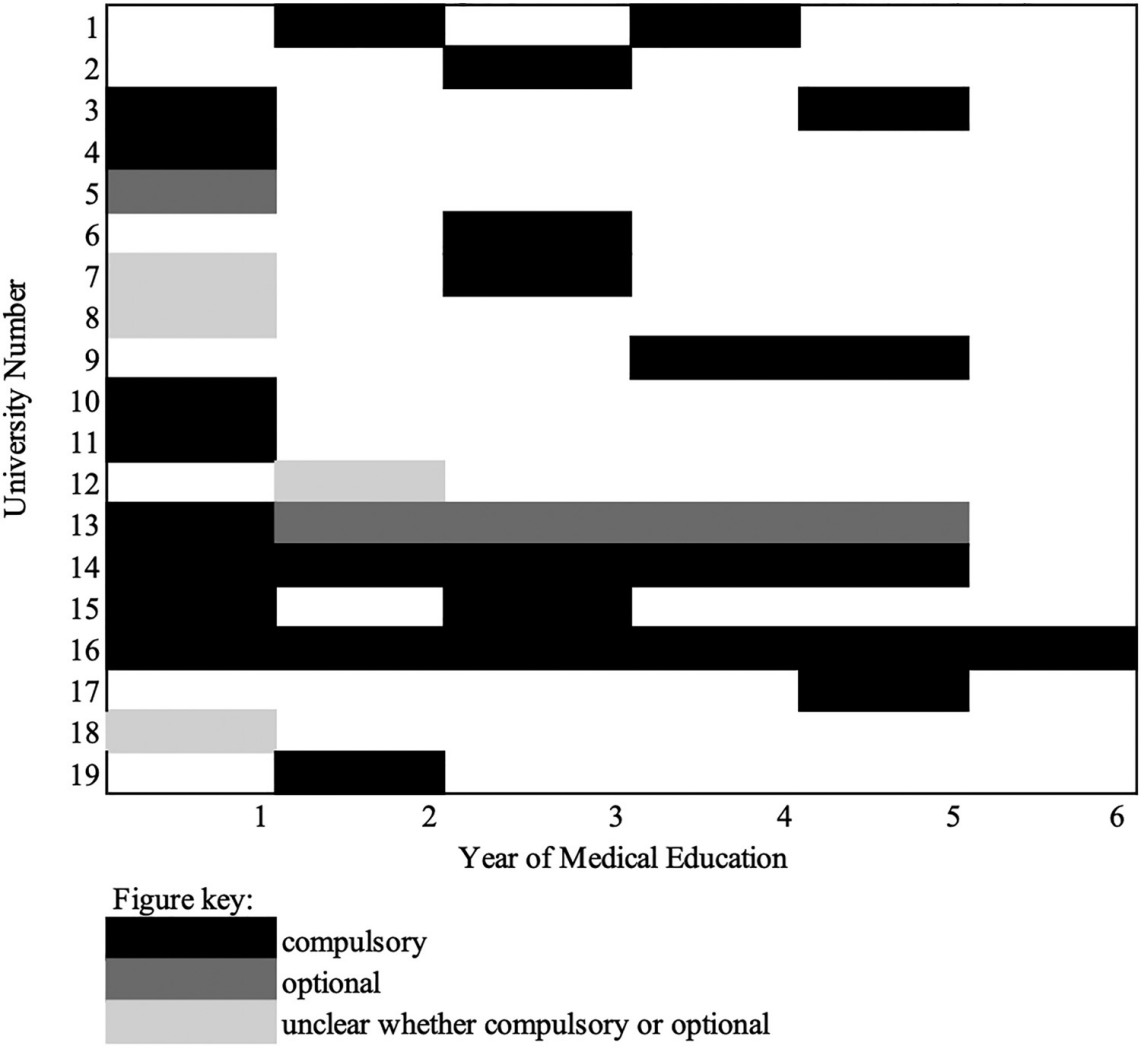
SH training schedule for UK medical universities (*n* = 19).

Half of the UK's public medical universities identified that some of their training was compulsory in nature (*N *= 17). Universities identified 35 training opportunities in total in their responses, and of these 29 instances were said to be compulsory, while six were optional to students. Some of these training opportunities were spread across multiple years of the course (see Figure 1). Figure 1 only shows the training schedule for UK medical universities that provided information on the year of such trainings, as not all universities provided all the data promptly or in the format requested, even in response to follow up requests, common issues reported around FOI requests made in the UK.^18^

**Figure 2.**
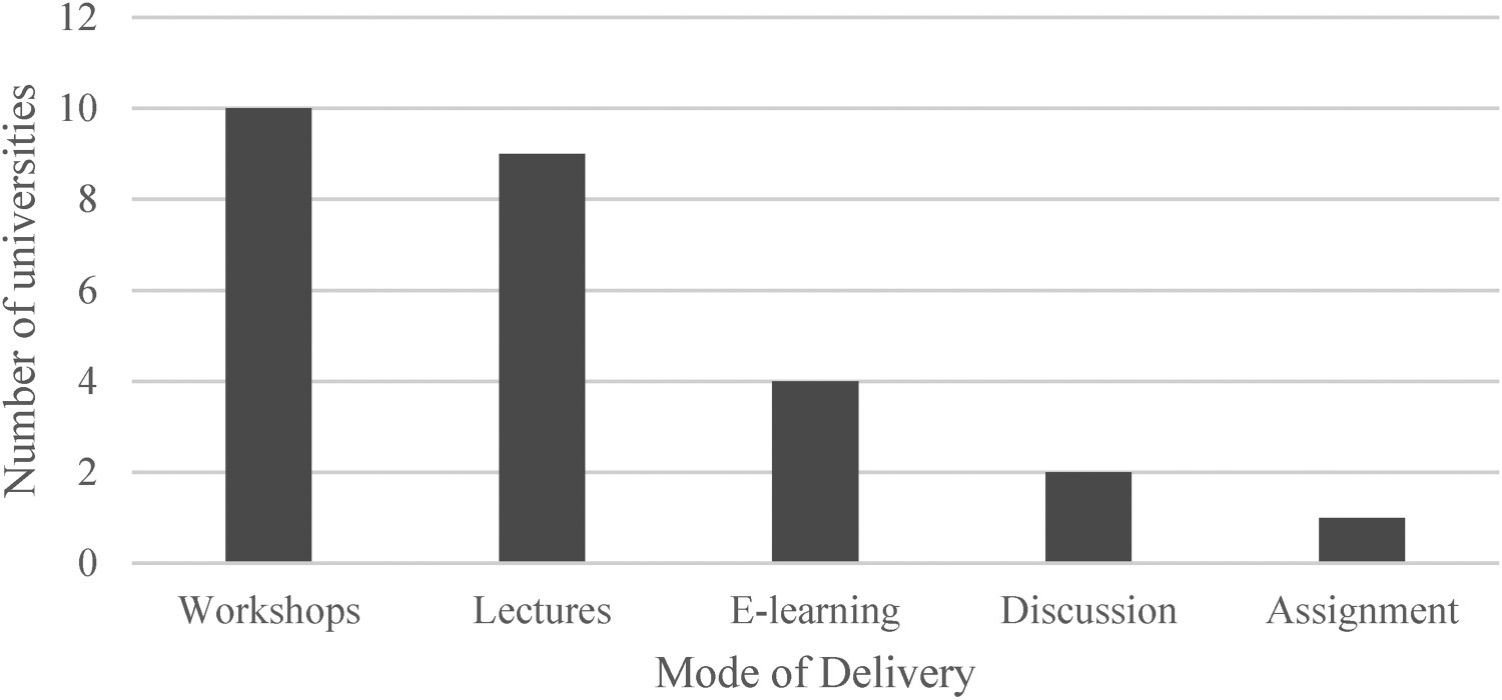
Number of universities that use each delivery method (*N* = 16).

A mix of methods of delivery also came through in the responses. Of the schools that detailed delivery type (*N* = 16), 10 universities used workshops, 9 used lectures, 4 used e-learning, 2 used discussions, and one used assignments (Figure 2). Six universities combined methods of delivery to include two or more of these approaches.

**Figure 3.**
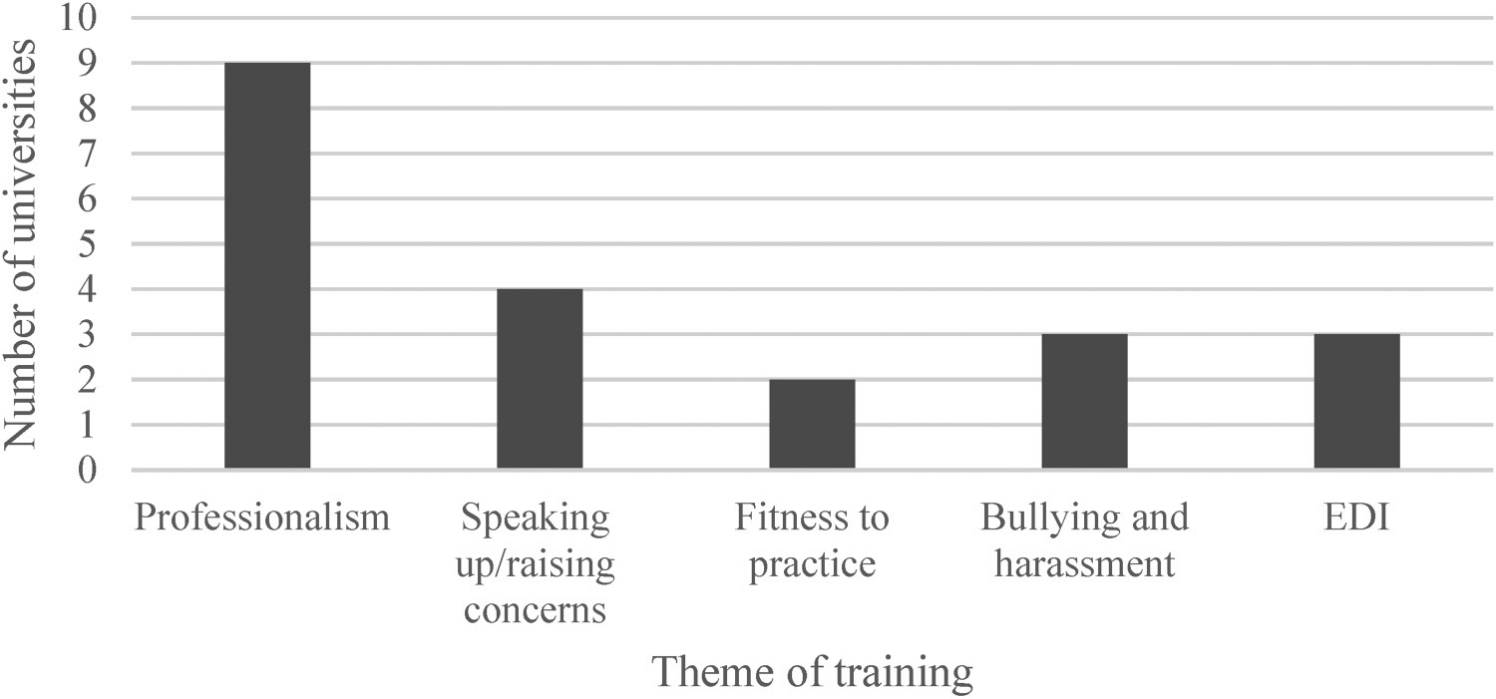
Number of universities that use each theme of training (*N* = 16).

Notably, only 16 of the universities included information about the theme of training. The most common ways in which the training was presented by these schools was under professionalism training, often with reference directly to GMC guidance (Figure 3). Other schools ran training as part speaking up and raising concerns training, fitness to practise training, bullying and harassment trainings, or training on Equality, Diversity, and Inclusion (EDI). Four schools presented sexual harassment training under two or more different themes.

Data on the percentages of students who attended training were not consistent across university responses. Some universities reported the raw number of students who attended, while others simply indicated that “all” students attended or reported that the training was compulsory. As many universities offered multiple trainings and were inconsistent in how data were provided in response to the FOI, while other responses lacked attendance data entirely, we were unable to determine percentage of those who attended trainings.

Of the universities that did not provide any training, only two of these schools detailed that they were considering such education. For these schools, building on existing curricula might be made difficult by an unwillingness to share best practice. Notably, four of the institutions that offer training refused the request for training content on the basis of needing to be competitive with other clinical schools. One public institution denied the request on the basis of:

ensuring that the Medical School, which is partially funded by public monies, maintains its competitive edge against other institutions in order to produce the best graduates who will become the next generation of doctors.

Another refused the information, suggesting:

the information would enable our competitors to have insight into the University's strategies and investments in the field of student recruitment upon which our marketing strategy is based.

Another stated:

if disclosed, would enable other institutions to adapt their courses to gain a competitive advantage in the recruitment of students.

## Discussion

The responses identify no standardization of training on sexual misconduct across medical universities in the UK. Almost half of schools (47 percent, *N *= 16) offered no training, or only generalised harassment training that was not specific to sexual misconduct or that was wholly outside the context of being a doctor. In the remaining 53 percent of schools where training was provided, a majority commendably placed the training into modules that were compulsory, although a wide range of delivery methods were adopted, with lectures and workshops being the main delivery approach. Training was most often given in professionalism modules, although it was sometimes present in speaking up and raising concerns, fitness to practise, bullying and harassment, and EDI modules. With such significant variations in the context and format of teaching when it was delivered, we therefore suggest that it is important to research which methods and content are most effective in improving these futures clinicians’ responses to this form of abuse and discrimination. This will help medical schools adopt evidence-based techniques that are both effective and consistent across the UK when delivering sexual harassment education.

Notably, in many cases, universities referenced professionalism training based on the GMC guidelines. The training cited was often in context to profession boundaries, fitness to practice, and good medical practice. This suggests professional guidance from professional organizations plays a central role to motivate universities to include it in the curriculum. However, such guidance must be written so as to ensure that it is complemented with content on how to respond, equipping students with skills, building a comprehensive education rather than providing content that restates policies and encourages mere compliance. We note the GMC content provides standards of conduct but does not include comprehensive skills-focused content that equips people will intervention techniques or options for addressing behaviours in the clinical setting. We note that such applied skills maybe achieved through use of scenario-based learning, active bystander training, and simulation, amongst other options.^1^ As such, the evolution of best practice around training curriculum requires medical educators to share knowledge and explore different models of delivery, including engaging in research longitudinally on curriculum impacts once medical students move on to be clinicians. Further studies should be performed to examine the different characteristics of the training programs currently being offered at universities and evaluate their effectiveness.

Notably, our curriculum analysis was significantly hampered by several schools refusing to provide curriculum on the basis of it being proprietary knowledge. The idea that public universities offering medical education in accordance with the GMC requirements are in competition such that they do not share curricula and do not engage in knowledge exchange is concerning. Such views on competition could obstruct analysis of best practice and also impede meaningful sharing knowledge and experience around module delivery, sequence of delivery, and effectiveness of training. It is unclear how schools also plan to differentiate their curricula on sexual misconduct and market it as exceptional and non-standard, especially considering the clear connection to good medical practice and equality, diversity, and inclusion.

Beyond the refusal to provide curricula, our study was also limited in several other ways. As with all studies using freedom of information requests, there is a potential for misinterpretation of the responses. To minimize the risk, several mitigation techniques were employed. We engaged careful phrasing in the original request to distinguish sexual harassment training from teaching on delivering patient care victim-survivors of harassment and violence. The request encouraged universities to seek clarification. Any responses received that were unclear or did not properly address the question were followed up with an email to clarify how it should be interpreted. Still the coded process may have introduced bias. However, all responses are public information and therefore the public or other research teams are able to access the data. That said, there is also a possibility that the responses provided by the universities were not complete or were inaccurate. It is known that survey respondents may provide responses that are considered more desirable or in line with social norms, and we note some schools described other auxiliary training that was not the target of this study, while not specifying that they did not offer medical training. Also, this study only examined training in UK public medical schools, and thus the findings are only applicable to the study population and cannot be generalised to other countries. As it is important to understand what training future NHS doctors may have before entering the workforce, it is important to note that many foreign-born and overseas trained doctors work in the NHS,^19^ and thus future studies should also explore training around the world.

## Conclusion

Here, we observe that graduates from more than one-third of schools in the UK are leaving their medical training without being educated on sexual misconduct and the medical profession anywhere in their degrees. It cannot be assumed, therefore, that graduates who are working as junior doctors have received training on sexual misconduct before starting their NHS roles. Understanding workplace training in healthcare workplaces like hospitals and general practice settings, as well as training in deaneries—the bodies in the UK that oversee post-graduate medical training within a region for core and specialty training—is critical to understand whether medical professionals, including both UK and foreign-trained graduates, are receiving adequate training to prevent sexual misconduct and equip people to respond appropriately and intervene when it happens. With costs in damages to address sexual misconduct in the NHS exceeding £4 million in the last five years, a comprehensive response across education and the health service is needed.

However, just as sexual misconduct and other forms of gender-based violence are not issues confined to one country, our responses require international cooperation and cross-national learning. It is essential that innovations in teaching and learning are shared both within countries and between countries, as university-based and national curriculum developments can inform better educational practice around the world. Responses to encourage collaboration and cross-learning globally, and to facilitate the creation of broader and more effective approaches in the educational and clinical settings, must be implemented.

In sum, medical students, as future clinicians, have a crucial and strategic need for education that allows them to perform a critical role in exhibiting good behaviour, and intervening, identifying, assessing, and reporting sexual misconduct when they see it happening at work or in wider society. Despite a renewed focus on this subject in recent decades, there are serious shortcomings within the health sector in preventing and addressing sexual misconduct. As such, we recommend expanding education on sexual misconduct within UK medical schools to establish the foundation for best practice in future clinicians, alongside critical skills that allow individuals to recall how they might intervene when they see sexual harassment in the future in healthcare settings.
